# Intricacies Affiliated With Post-COVID Vaccine Complications in Makkah Province, Saudi Arabia

**DOI:** 10.7759/cureus.32749

**Published:** 2022-12-20

**Authors:** Muazzam M Sheriff, Samaher G Basalib, Maya J Mereani, Layali M Bakhsh, Bayan A Alzamzami, Raha M Garout

**Affiliations:** 1 Medicine, Ibn Sina National College for Medical Studies, Jeddah, SAU

**Keywords:** coronavirus disease 2019 (covid-19), post-covid 19 vaccine complications, pfizer-biontech, moderna, minor side effects, major side effects, makkah province, astrazeneca

## Abstract

Introduction

COVID-19 vaccine side effects have a fundamental role in public confidence in the vaccine and its uptake process. Thus far, evidence on vaccine safety has been exclusively obtained from manufacturer-sponsored studies; therefore, this study is designed to assess post-COVID-19 vaccine complications in Makkah province, Saudi Arabia.

Method

A cross-sectional study included 840 subjects conducted from August to November 2022 to collect data about COVID-19 vaccine side effects. A validated questionnaire was used with 21 multiple-choice items covering demographic data, COVID-19 vaccination type, side effects, and medication used to relieve side effects. The online Raosoft sample size calculator (Raosoft Inc., Seattle, Washington) was utilized for sample size calculation. The Statistical Package for the Social Sciences version 22.0 (IBM Inc., Armonk, New York) was utilized to carry out descriptive statistics. The Shapiro-Wilk test was used to evaluate normal data distribution. Significance of categorized data made by the Pearson's Chi-Squared test and of the vaccination side effects experienced in general wellbeing on a scale of 1-10 by the Kruskal-Wallis test.

Result

The survey found that most participants in the 18-26 age group were from Jeddah, women, and Saudis. Only 7.1% of participants had comorbidities alone, and 63.1% of participants had previously had COVID-19. The vaccine types used in this study were primarily Pfizer (83.3%), AstraZeneca (9.5%), Moderna (3.6%), and combination vaccines (3.6%). The majority of the participants received vaccination up to the third dose. No side effects were reported by 9.5% of participants, while mild and severe side effects were reported by 90.5% and 23.8% of participants, respectively. Mild side effects included injection site pain, redness, tenderness, or itching (34.5%), fatigue (22.6%), low-grade fever, chills, diarrhea, headache (17.9%), and myalgia (14.3%).

Conclusion

The majority of the side effects of COVID-19 vaccination were minor reactions (90.5%), but 23.8% were found to be serious side effects, most of which lasted one to three days. More independent studies are needed to investigate gender differences, COVID-19 vaccine efficacy, and the prevalence of side effects in other populations conducted by academic institutions. Additional independent research on vaccine safety is urgently needed to increase public confidence in vaccines and to better understand risk factors for vaccine side effects.

## Introduction

SARS-CoV-2, which causes COVID-19, is one of the greatest issues the world is currently facing. It resulted in financial losses, societal repercussions, and a significant burden on the healthcare system [1]. On March 11, 2020, SARS-CoV-2 was declared a global pandemic [2]. The only reliable preventive approach before COVID-19 vaccinations introduction was to limit exposure. Social isolation, curfews, and widespread economic shutdowns all negatively affected people's physical and mental health and the global economy [3]. Even if a variety of therapeutic drugs have been proposed to combat COVID-19, their efficacy and potency must be determined by additional randomized control studies [4]. The greatest method for limiting the pandemic is immunization against COVID-19. Within a year following the initial reports of COVID-19, vaccinations were created thanks to the tireless efforts of governments and scientists, as well as thanks to advanced biotechnology and interim studies [5]. Several contender COVID-19 vaccines were created using a variety of platforms. The general public's post-vaccination experience is still little understood.

On April 1, 2022, the World Health Organization (WHO) situation report stated that there were over 486 million illnesses and around 6.14 million deaths worldwide. Over 62 million doses of the COVID-19 vaccine were provided, and there were officially verified cases of 750,814 illnesses and 9045 deaths in Saudi Arabia [6]. The population's perceptions of the advantages and hazards of vaccines, as well as their trust in vaccination, as a result, have a role in the effectiveness of vaccination efforts. Lack of awareness regarding the immunization's relative benefit-to-risk ratio, according to researchers, is the cause of vaccination refusal or postponement [7,8]; this is a condition that the WHO Strategic Advisory Group of Experts on Immunization identified in 2015 as vaccine hesitancy (VH) [9]. Moreover, in 2019, WHO identified this hesitation as one of the most significant dangers to world health [10]. According to Mevorach et al., much of the material that is accessible about the COVID-19 vaccine's adverse effects was published by research that was supported by the manufacturers, which are following drug authorities' guidelines and was monitored by third parties [11]. Therefore, the acceptance of vaccinations could be negatively impacted by the absence of independent research on their safety, which must be increased in the coming months in order to break the virus and its variants out of this vicious cycle [12].

As with any routine treatment, vaccination might have unintended consequences or side effects in certain patients [13]. Adverse early effects with any vaccination are common and can range from local side effects, such as swelling and pain, to systemic ones, such as nausea, headache, chills, fatigue, myalgia, and fever [14]. One profound side effect that could happen is anaphylaxis to vaccine constituents, which was thought to originate from polyethylene glycol (PEG) allergic reactions [14]. Additionally, it was hypothesized that the injection of COVID-19 vaccines, including those made by AstraZeneca, Pfizer, and Moderna, resulted in blood clotting episodes [15,16].

So, the aim of this research was to determine how common adverse effects of the COVID-19 vaccine were delivered to residents of Makkah Province, Saudi Arabia.

## Materials and methods

Study design, setting, and participants

A cross-sectional online survey study was made in the Saudi province of Makkah with participants who had received any of the provided vaccinations (Pfizer, AstraZeneca, Moderna, and Mix). Data were collected between August and November of 2022. People using social networking sites were randomly selected to complete a self-administered online survey created on Google Forms (Google, Mountain View, California) for the study (email, Facebook, and WhatsApp). The study utilized a self-administered questionnaire. A webpage containing a brief summary of the study's goals and directions for completing the survey was given to prospective participants. Each respondent was sent a standard general invitation letter with the survey link and a statement that participation was optional and that all responses would be kept anonymous in order to get their informed consent prior to the data collection. Participants were not allowed to access the survey or take part in the study if they rejected consent. People who followed the link were taken to Google Forms, where they had to complete all of the questions in order to move on to the survey's next portion and prevent concerns with missing data. The study was conducted under the title "Intricacies Affiliated Post Covid Vaccine Complications in Makkah Province KSA" and was approved by Ibn Sina National College Research Review Board Institutional Human Ethics Committee with ethical approval IRRB-01-2011-2022 along with the protocol identification number 005MP14102022. Out of 850 surveys received from respondents, 840 participants aged ≥18 years were included in this research. In addition, participants who received COVID-19 immunization were included in the study. A person must be at least 18 years old, vaccinated, and a Makkah resident to participate.

Survey and data collection

Multiple-choice survey questions were offered in both English and Arabic. A team of professionals evaluated the survey's many questions in order to validate it. The survey consisted of two parts. The first part consisted of seven demographic and clinical characteristics questions about residence region, age, gender, nationality, education level, presence of chronic comorbidities, and previous infection with COVID-19. There were a series of questionnaires related to the side effects providing a detailed analysis in the second section of this cross-sectional study. The participants were also asked about medical assessment and medications used after minor and major complications and if they completely recovered after mediation, the number of days to recover after medical assistance, as well as vaccination side effects experienced in general well-being on a scale of 1-10. Survey reliability is assessed using the internal consistency test known as Cronbach's alpha. The survey tool's overall dependability was 0.81, indicating that it was trustworthy and had good internal consistency [17].

Sample size

The online Raosoft sample size calculator (Raosoft Inc., Seattle, Washington) was utilized for sample size calculation. According to the world meter elaboration of the latest United Nations data, the Makkah province is about 2.115 million [2]. We suggested that the confidence level is 95%, the error margin is 3%, and the response distribution is 50%. The sample size recommended was 385; the study included 840 participants who completed the questionnaire.

Statistical analysis

The Statistical Package for the Social Sciences version 22.0 (IBM Inc., Armonk, New York) was utilized to carry out descriptive statistics. The Shapiro-Wilk test is used to evaluate normal data distribution. Data are presented as frequencies and percentages for categorized data and mean and standard deviation (SD) (minimum and maximum) for parametric data. Significance of categorized data made by Pearson's Chi-Squared test and of the vaccination side effects experience in general well-being on a scale of 1-10 by Kruskal-Wallis test as data were not normally distributed.

## Results

The majority of the participants were from Jeddah (88.1%), then Makkah (4.8%), and other places (7.1%), with significant differences between them p<0.0001. Female responders (64.3%) were significantly higher than males (35.7%), with significant differences between them p=0.009, and Saudis (82.1%) were significantly higher than non-Saudis (17.9%), with significant differences between them p<0.0001. The number of participants in the age group 18-26 years (41.7%) was significantly higher than in other age groups, with significant differences between them p<0.0001. Only 7.1% of the participants had comorbidities, and 63.1% of the participants were previously infected with COVID-19 (Table 1).

**Table 1:** Socio-demographic characteristics of all participants (n=840)

Socio-demographic characteristics	Response rate	p-value significance
Place of residency		0.0001
Jeddah	88.10%	
Makkah	4.80%	
Others	7.10%	
Gender		0.009
Male	35.70%	
Female	64.30%	
Age group		0.0001
18-26 years	41.70%	
27-36 years	17.90%	
37-46 years	16.70%	
47 -56 years	8.30%	
> 56 years	15.50%	
Nationality		0.0001
Saudi	82.10%	
Non-Saudi	17.90%	
Education level		0.004
Undergraduate	21.40%	
Graduate	41.70%	
Post-graduate	21.40%	
Doctorate	15.50%	
Comorbidities		0.0001
No	92.90%	
Yes	7.10%	
Previously infected with COVID-19		0.016
No	36.90%	
Yes	63.10%	

**Figure 1:**
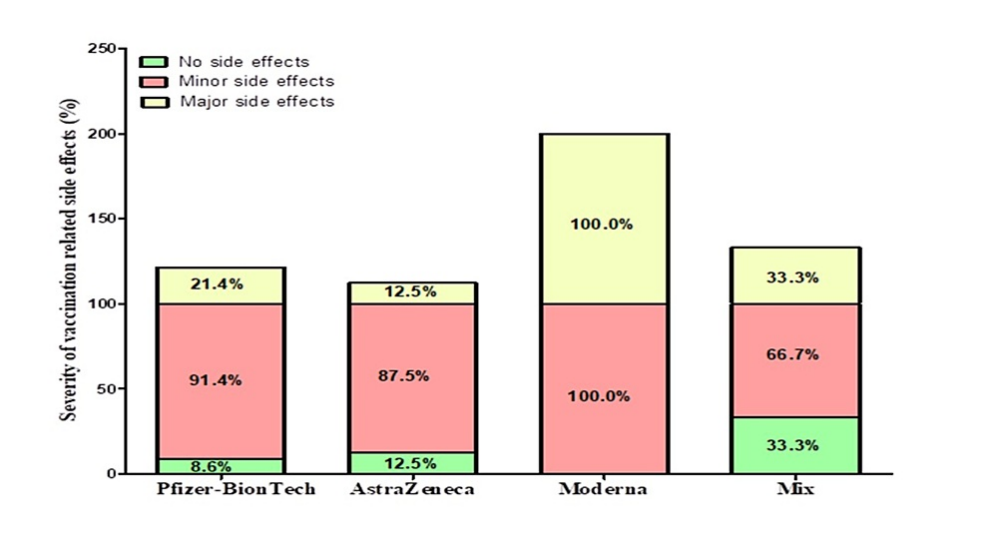
Severity of side effects

The type of vaccine used was mostly Pfizer-BioNTech (Comirnaty®) (n=700, 83.3%), then AstraZeneca (Vaxzevria®, COVISHIELD™) (n=80, 9.5%), Moderna (Spikevax®) (n=30, 3.6%), and mix (n=30, 3.6%). This cross-sectional study showed that a mere 2.4% of the participants received only a single dose of vaccination. 85.7% of the participants in this survey received three doses of vaccination, 8.3% of the participants in this survey received two doses of vaccination, whereas 3.6% of the participants received vaccination up to the fourth dose. No side effects were reported in 9.5% of participants. The majority of the participants were from Jeddah (88.1%), then Makkah (4.8%), and other places (7.1%), with significant differences between them p<0.0001. Female responders (64.3%) were significantly higher than males (35.7%), with significant differences between them p=0.009, and Saudis (82.1%) were significantly higher than non-Saudis (17.9%), with significant differences between them p<0.0001. The number of participants in the age group 18-26 years (41.7%) was significantly higher than in other age groups, with significant differences between them p<0.0001. Only 7.1% of the participants had comorbidities, and 63.1% of the participants were previously infected with COVID-19 (Table 1). While minor and major side effects were reported in 90.5% and 23.8% of participants, respectively. Minor side effects, including pain at the injection site, redness, tenderness, or itching, was experienced among 34.5% of participants, while 22.6% of participants experienced fatigue and tiredness. Minor side effects like low-grade fever, chills, diarrhea, and headache were encountered among 17.9% of participants, while 14.3% of participants experienced muscle pain. The major side effects of high-grade fever was experienced among 15.5% of participants, with 6% of the participants experiencing anxiety, while 2.4% of participants experienced shortness of breath. The hypersensitivity allergic reactions due to the vaccination were reported by 9.5% of participants. The percentage of minor and major side effects reported by each vaccine showed in Figure 1. The allergic reactions were reported both in Pfizer-BioNTech and Moderna vaccines. The timeline after which side effects were experienced was mostly 12-24 hours of vaccination (47.6%), then <12 hours of vaccination (25%), and lastly, 24-48 hours of vaccination (17.9%) (Table 2).

**Table 2:** Immunization details and side effects after receiving vaccines related to vaccine type

Data	Total (n=840)	Pfizer-BioNTech (n=700)	AstraZeneca (n=80)	Moderna (n=30)	Mix (n=30)	p-value significance
Immunization details						0.004
Single dose	2.4%	1.44%	-	33.3%	-	
Double dose	8.3%	8.6%	12.5%	-	-	
Triple dose	85.7%	85.7%	87.5%	66.7%	100%	
Four doses	3.6%	4.3%	-	-	-	
Minor side effects						0.483
Pain at injection site, redness, tenderness, or itching	34.5%	37.1%	12.5%	66.7%	-	
Fatigue and tiredness	22.6%	22.9%	37.5%	-	-	
Low-grade fever, chills, diarrhea, and headache	17.9%	18.6%	12.5%	-	33.3%	
Muscle pain	14.3%	12.9%	25%	-	33.3%	
None of above	10.7%	8.6%	12.5%	33.3%	33.3%	
Major side effects						0.145
High-grade fever	15.5%	12.9%	12.5%	66.7%	33.3%	
Anxiety	6%	5.7%	-	33.3%	-	
Shortness of breath	2.4%	2.9%	-	-	-	
None of above	76.2%	78.6%	87.5%	-	66.7%	
Allergic reactions						0.008
Skin burning	3.6%	2.9%	-	33.3%	-	
Rash	3.6%	4.3%	-	-	-	
Red welts (raised bumps) on face and lips	2.4%	1.4%	-	33.3%	-	
None of above	90.5%	91.4%	100%	33.3%	100%	
Timeline after which the side effects were experienced						0.455
None	9.5%	8.6%	12.5%	-	33.3%	
<12 hours of vaccination	25%	22.9%	50%	33.3%	-	
12-24 hours of vaccination	47.6%	47.1%	37.5%	66.7%	66.7%	
24-48 hours of vaccination	17.9%	21.4%	-	-	-	

10.7% of the participants procured medical assistance due to minor side effects. The minor side effects distribution among the vaccination were 7.1% for participants vaccinated with the Pfizer-BioNTech vaccine, 25% for participants vaccinated with the AstraZeneca vaccine, and 66.7% for participants vaccinated with the Moderna vaccine. 8.9% of the participants procured medical assistance due to major side effects. The major side effects distribution among the vaccination were 4.3% for participants vaccinated with the Pfizer-BioNTech vaccine, 25% for participants vaccinated with the AstraZeneca vaccine, and 66.7% for participants vaccinated with the Moderna vaccine. 57.1% of participants took medications to treat minor side effects. The distribution of vaccines based upon the medications taken to treat minor side effects were as follows: 54.3% of participants vaccinated with the Pfizer-BioNTech vaccine, 75% of participants vaccinated with the AstraZeneca vaccine, 100% of participants vaccinated with the Moderna vaccine, and 33.3% with mixed vaccines. 41.7% of participants took medications to treat major side effects. The distribution of vaccines based upon the medications taken to treat minor side effects were as follows: 17.1% of participants vaccinated with the Pfizer-BioNTech vaccine, 25% of participants vaccinated with the AstraZeneca vaccine, and 100% of participants vaccinated with the Moderna treated minor side effects with medications. This study showed that a cumulative average total of 45.2% of participants recovered completely after seeking medical assistance post-vaccination complications. The data percentage for each vaccination out of 45.2% participants reported complete recovery after seeking medical assistance was as follows. 40% of participants who recovered completely after seeking medical assistance were vaccinated with the Pfizer-BioNTech vaccine, 62.5% of participants vaccinated with the AstraZeneca vaccine, and 100% vaccinated with the Moderna vaccine. 66.7% vaccinated with mixed vaccine showed a complete recovery after seeking medical assistance. In this study, 41.7% of the participants, after seeking medical assistance, recovered the same day, 31.0% of the participants took a couple of days to recover, and 27.4% of the participants took more than two days for their recovery. The mean of vaccination side effects experienced in general wellbeing on a scale of 1 to 10 was 4.31 for all participants, with the highest in the Moderna vaccine and lowest in the mixed vaccine, with significant differences between the groups being p=0.414 (Table 3).

**Table 3:** Medications used after vaccination related to vaccine type

Medications	Total (n=840)	Pfizer-BioNTech (n=700)	AstraZeneca (n=80)	Moderna (n=30)	Mix (n=30)	p-value significance
Medical assistance due to minor side effects						0.005
No	89.3%	92.9%	75%	33.3%	100%	
Yes	10.7%	7.1%	25%	66.7%	-	
Medical assistance due to major side effects						0.0001
No	91.7%	98.7%	75%	33.3%	100%	
Yes	8.9%	4.3%	25%	66.7%	-	
Medications taken to treat minor side effects						0.239
No	42.9%	45.7%	25%	-	66.7%	
Yes	57.1%	54.3%	75%	100%	33.3%	
Medications taken to treat major side effects						0.004
No	79.8%	82.9%	75%	-	100%	
Yes	20.2%	17.1%	25%	100%	-	
Complete recovery after medical assistance						0.399
None	41.7%	45.7%	25%	-	33.3%	
No	13.1%	14.3%	12.5%	-	-	
Yes	45.2%	40%	62.5%	100%	66.7%	
Days to recover after medical assistance						0.806
None	41.7%	44.3%	37.5%	-	33.3%	
1-2 days	31.0%	28.6%	37.5%	66.7%	33.3%	
>2 days	27.4%	27.1%	25%	33.3%	33.3%	
Vaccination side effects experience in general wellbeing on a scale of 1 to 10	4.31±2.32 (1-10)	4.41±2.26 (1-9)	3.38±2.07 (1-7)	5.67±4.04 (2-10)	2.50±2.12 (1-4)	0.414

## Discussion

The findings of this study show that the vast majority of participants were from Jeddah, females, and Saudi, in the age group 18-26 years. A mere 7.1% of participants only had comorbidities, and 63.1% of participants were previously infected with COVID-19. The types of vaccine used in this study were mainly Pfizer (83.3%) then, AstraZeneca (9.5%), Moderna (3.6%), and mixed vaccine (3. 6%). This cross-sectional study showed that a mere 2.4% of the participants received only single dose of vaccination. 85.7% of the participants in this survey received three doses of vaccination, 8.3% of the participants in this survey received two doses of vaccination, and 3.6% of the participants received vaccination up to the fourth dose.

There were no side effects reported in 9.5% of participants, while minor and major side effects were reported in 90.5% and 23.8% of participants, respectively. Minor side effects were pain at the injection site, redness, tenderness, or itching (34.5%), fatigue and tiredness (22.6%), low-grade fever, chills, diarrhea, and headache (17.9%), and muscle pain (14.3%). The major side effects were high-grade fever (15.5%), then anxiety (6%), and lastly, shortness of breath (2.4%). The timeline after which side effects were experienced was mostly 12-24 hours after vaccination (47.6%), then <12 hours after vaccination (25%), and lastly, 24-48 hours after vaccination (17.9%). Several studies have assessed post-vaccination adverse reactions of the Pfizer, Moderna, and AstraZeneca vaccines [18-22].

Different COVID-19 vaccine side effects studies have shown findings that are similar to ours [23,24]. A randomized controlled trial (RCT) that enrolled 43,000 volunteers with a median age of 52 years provided the first data to assess the effectiveness of the Pfizer COVID-19 vaccine. Vaccine efficiency was around 95%, according to the preliminary findings of this RCT, but some adverse side effects appeared a few days after the vaccination [11]. The side effects of immunization may be divided into local and systemic reactions, and their intensity ranges from mild to moderate [25]. Injection site pain, headaches, and exhaustion were listed as the three most frequent adverse effects (≥10%) in the Food and Health Bureau of Hong Kong's evaluation report on CoronaVac (an inactivated viral vaccine) [26]. Among Czech medical professionals, the Pfizer COVID-19 vaccine's most frequent side effects were soreness at the injection site, weariness, headache, muscle pain, chills, and joint pain [22]. In the study conducted among healthcare professionals in Turkey, it was discovered that more than 10% of participants reported experiencing injection site discomfort (41.5%), weariness (23.6%), and headache (18.7%) [23]. A study reported pain at the injection site, headaches, flu-like symptoms, fever, and fatigue are the most typical symptoms. Fast heartbeat, body aches, respiratory problems, joint pain, chills, and sleepiness are less frequent adverse effects. Bell's palsy and pain and swelling of the lymph nodes were uncommon side effects [18].

A study reported several side effects after taking the COVID-19 vaccination in the Jordanian population, chiefly flu-like symptoms, pain at the injection site, and gastrointestinal symptoms [27]. The number of side effects that were recorded was noticeably higher in individuals who received the AstraZeneca vaccine. Although vaccine reactogenicity appears to decline with aging and is correlated with a transient rise in inflammatory cytokines, it is not thought to be a reliable predictor of a favorable immune response [28]. Several studies have linked the administration of Pfizer or AstraZeneca vaccines to fatigue [23,24,29,30]. In addition, gastrointestinal symptoms, mainly nausea, were recorded by 23% of those who had taken the AstraZeneca vaccine. In contrast, lower proportions were seen with Pfizer vaccine administration in two cross-sectional studies (15.94% and 13.0%), as well as one randomized trial with Sinopharm vaccine administration (1%) [20,23]. Nausea was observed in a WHO report with AstraZeneca vaccine administration. Half of those who received the Pfizer or AstraZeneca vaccinations reported having headaches [20,23]. Two cross-sectional studies, including healthcare personnel who received the Pfizer vaccination, showed localized lymphadenopathy [23]. Dizziness was the only neurological symptom reported in the study. Other reported neurological manifestations were considered rare with the administration of the Pfizer vaccine [20], with less than a 1% prevalence of stress and depression.

Several reports on vaccine adverse reactions mention pain at the injection site [18,23]. Researchers advise lowering the patient's injected arm in order to lessen pain because injection into a relaxed muscle leads to less pain versus one that is tensed. Additionally, it's important to keep vaccines on-site at a low temperature. The likelihood of pain at the injection site may rise if the injection is performed without adequate warming [23]. A further factor in how painful the injection was may be muscle mass. To lessen disparities in patients' experiences of pain following vaccination, it is strongly advised that healthcare professionals who are involved in the process obtain the required training on the best injection techniques. The process of developing immunity following a vaccination might occasionally result in undesirable side effects.

Following first and/or second doses, the Pfizer COVID-19 vaccine may cause mild side effects, such as soreness, swelling or redness at the injection site, chills, fatigue, headaches, muscle and joint aches, and fever. These symptoms can be a sign that the body is constructing required defense immunity. This finding could be explained by the immune system's reaction. The immune system has the capacity to release cytokines that cause inflammation in the muscles, blood vessels, and other tissues. Additionally, it could manifest flu-like symptoms that last after vaccination for days. In this study, allergic reactions to the vaccine were reported in 80 participants of the 30 (3.6%) had skin burning, 30 (3.6%) had a rash, and 20 (2.4%) had red welts on their face and lips.

The allergic reactions reported in Pfizer and Moderna vaccines. Egg protein, gelatin, formaldehyde, thimerosal, or neomycin are examples of inactive ingredients that may contribute to allergic reactions to vaccines that are not directly linked to the active vaccine itself. These substances also play a role in certain immunoglobulin E-mediated immediate reactions. Excipients, which are inert compounds added to vaccines to improve stability, solubility, absorption, alter palatability, or produce a distinctive look, are defined by the European Medicines Agency as components of a medical form apart from the active ingredient. Excipients can result in a variety of clinical allergic responses, from skin conditions to potentially fatal systemic reactions. It has been suggested that the Pfizer COVID-19 vaccination may cause a serious allergic reaction.

After getting the vaccine dose, an acute allergic reaction will eventually occur within a few minutes to an hour. Dyspnea, swelling of the face and throat, a rapid heartbeat, a body rash, dizziness, and weakness can all be symptoms of an acute allergic reaction. Bell's palsy has also been listed as a relatively uncommon adverse reaction to the vaccination. In this study, medical assistance due to minor and major side effects was used in 10.7% and 8.9%, respectively. Medical taken to treat minor and major side effects used by 57.1% and 41.7%, respectively.

This study shown that a cumulative average total of 45.2% participants recovered completely after seeking medical assistance post vaccination complications. The data percentage for each vaccination out of 45.2% participants reported complete recovery after seeking medical assistance was as follows. 40% of participants who recovered completely after seeking medical assistance were vaccinated with the Pfizer-BioNTech vaccine while 62.5% participants vaccinated with AstraZeneca vaccine and 100% vaccinated with the Moderna vaccine. 66.7% vaccinated with mixed vaccine showed a complete recovery after seeking medical assistance.

In this study, 41.7% of the participants, after seeking medical assistance, recovered the same day, 31.0% of the participants took a couple of days to recover, while 27.4% of the participants took more than two days for their recovery.

The mean of vaccination side effects experienced in general well-being on a scale of 1 to 10 was 4.31 for all participants, with the highest in the Modena vaccine and the lowest in the mixed vaccine. Several studies found a similar pattern, with the majority of adverse effects being mild to moderate in severity and typically going away a few days after vaccination [24,30].

Limitations

The questionnaire was conducted online in the study, which raises the possibility that the study was subject to selection bias. Moreover, the cross-sectional design of this study that depends on self-assessed and self-reported outcomes is obviously a limitation. Another limitation of the study is that it was done in the Makkah providence only.

## Conclusions

Most side effects of COVID-19 vaccination are rather minor reactions (90.5%), while major side effects found in 23.8% persist in most cases for one to three days. The most common minor side effects were pain at the injection site, redness, tenderness, or itching, then fatigue and tiredness, low-grade fever, chills, diarrhea, headache, and muscle pain. Major side effects were high-grade fever, anxiety, and lastly, shortness of breath. All the side effects are revealed by medical assessment and treatment. The majority of the symptoms only persisted for a few days following immunization and were often not severe enough to warrant hospitalization. Further independent studies are required to explore the gender-based differences as well as the prevalence of COVID-19 vaccine effectiveness and side effects in other population groups to be carried out in academic institutions. To increase public trust in the vaccine and better understand the risk factors for vaccine side effects, additional research on vaccine safety is urgently needed.
